# Psychosocial Adjustment to Sex Reassignment Surgery: A Qualitative Examination and Personal Experiences of Six Transsexual Persons in Croatia

**DOI:** 10.1155/2014/960745

**Published:** 2014-03-25

**Authors:** Nataša Jokić-Begić, Anita Lauri Korajlija, Tanja Jurin

**Affiliations:** Department of Psychology, Faculty of Humanities and Social Sciences, University of Zagreb, Ivana Lucica 3, 10000 Zagreb, Croatia

## Abstract

In Croatia, transgender individuals face numerous social and medical obstacles throughout the process of transition. The aim of this study was to depict the factors contributing to the psychosocial adjustment of six transsexual individuals living in Croatia following sex reassignment surgery (SRS). A combination of quantitative and qualitative self-report methods was used. Due to the specificity of the sample, the data were collected online. Standardized questionnaires were used to assess mental health and quality of life alongside a series of open-ended questions divided into 4 themes: the decision-making process regarding SRS; social and medical support during the SRS process; experience of discrimination and stigmatizing behaviors; psychosocial adjustment after SRS. Despite the unfavorable circumstances in Croatian society, participants demonstrated stable mental, social, and professional functioning, as well as a relative resilience to minority stress. Results also reveal the role of pretransition factors such as high socioeconomic status, good premorbid functioning, and high motivation for SRS in successful psychosocial adjustment. During and after transition, participants reported experiencing good social support and satisfaction with the surgical treatment and outcomes. Any difficulties reported by participants are related to either sexual relationships or internalized transphobia. The results also demonstrate the potentially protective role that a lengthier process of transition plays in countries such as Croatia.

## 1. Introduction

Studies have demonstrated a high prevalence of mental health problems among transsexual individuals (TS) [1–3]. Mental health problems in TS individuals are related to social environments which foster isolation, marginalization, a lack of social support, discrimination including interpersonal and systemic microaggression, and victimization [4, 5]. The link between societal stigma and discrimination and mental health problems is conceptualized in the Minority Stress Model proposed by Meyer [6]. According to this model, minority stress is stress that stems from being part of a minority and, as such, adds to the usual level of general stress experienced by all people. Because it is the result of social structures and norms that exist independently of the individual, it is chronic and is socially conditioned [7]. This framework suggests that stigma, prejudice, and discrimination create a hostile social environment, which causes internalized transphobia and mental health problems including depression, anxiety, and posttraumatic stress [7]. The findings also indicate that the rejection TS that individuals face is significantly more severe than the discrimination experienced by LGB individuals [8]. Research has demonstrated that social support, self-acceptance, and integration of minority identity can ameliorate minority stress [7]. Improving access to mental health and social services that affirm* trans* identity and promote resilience are noted as important preventive activities that can decrease minority stress and its negative consequences for the psychological and physical health of* trans* individuals.

According to the European Parliament [9], choosing one's gender or sex is a basic human right of every person. The Parliament thus calls on the Commission and the World Health Organization to withdraw gender identity disorders from the list of mental and behavioral disorders and to ensure a nonpathologizing reclassification in the negotiations for the 11th version of the International Classification of Diseases ICD-11. Professional opinion in western countries has shifted from transsexualism to transgenderism in the last two decades [10], in which a binary view on gender has given way to the notion of gender identity as a continuum, with male and female gender identities standing at each of its poles. This shift in expert opinion, in which gender nonconformity is seen as a gender variation rather than a disorder, has no doubt contributed to the psychological welfare of individuals undergoing sex reassignment surgery (SRS) and their families [10]. However, even in those countries representing benchmarks of good practice in the care of TS individuals, not all mental health experts adopt the same approach towards supporting transgender people [11].

Furthermore, although there are specialized centers providing multidisciplinary care for gender-dysphoric individuals in many countries, there are countries in which such forms of care are not available. Therefore, the question remains as to how far these most recent high standards of care for TS individuals can be interpreted and applied in culturally diverse countries with more traditional values. Indeed, in communities with more traditional values, the challenges faced by TS individuals differ from those faced by TS persons in urban western societies with a prescribed standard of care [12, 13]. In such societies, those who do not express their sexuality or gender in traditionally accepted ways may feel unwelcome [14]. In such environments, TS persons are met not only with incredulity, a lack of understanding, and stigmatization from their social environment, but also with the ignorance of the very experts from whom they are seeking support [15].

A final challenge arises in light of the vast availability of information via the Internet. With access to information about levels and standards of care across countries, TS individuals worldwide are now fully aware of the form and standard of care to which they are entitled, but which they are perhaps denied due to political, financial, geographical, or ideological contexts. This leads to high levels of frustration and impatience among TS persons, which might contribute to the decision to undergo very risky hormonal self-medication.

Croatia is a central eastern European country belonging to a group of European countries that show pronounced homonegative views [16]. In the past year, a right-wing political movement became very actively and visibly outspoken against homosexual marriage and gender ideology in a campaign that culminated in a national referendum decision on December 2013 to outlaw same-sex marriage. Attitudes towards gender-variant persons are rarely researched in Croatia, but the available data demonstrate that public attitudes are predominantly negative [17]. Consistent with this attitude is the complete absence of specialized care centers for TS persons in the Republic of Croatia and a notable lack of qualified mental health providers working with transgender persons [15]. Experts who work with TS individuals are small in number and connect with one another only on an informal basis, in most cases through TS individuals themselves. These TS individuals, in turn, share information via the Internet and, in such a way, are able to connect with experts who are transgender friendly and therefore are more knowledgeable about transsexuality. In Croatia, no accepted standards exist that would regulate diagnosis, therapy, and ethical questions pertaining to the care and support of TS individuals. Such standards would help both experts and TS persons in offering and organizing the necessary treatment [18]. Paradoxically, while the health care system does not provide appropriate treatment for TS persons, the Croatian legal system allows for the change of name and gender following SRS.

In this situation, TS persons are left to fend for themselves or solely in the hands of whichever doctor they come in contact with. Medical students and young doctors, during periods of study and residency, often do not receive comprehensive training regarding different gender variations or sexual orientations. In contrast, psychology students receive large amount of information about transgenderism. A small number of experts working with TS persons are attempting to further change the attitudes of their colleagues through lectures at professional conferences and by publishing articles in professional periodicals [18]. However, due to the hierarchical organization of the Croatian health care system, which positions doctors as superior to psychologists, this knowledge does not influence everyday clinical practice as much as it should.

Due to this lack of knowledge, doctors adopt a pathologizing approach towards TS individuals, treat transsexuality as a psychiatric disorder, and typically refer TS persons to psychiatric treatment. The knowledge and attitude of the mental health professionals with whom TS individuals come into contact then determine which steps are taken, including both the duration of psychotherapy and whether an endocrinologist or surgeon is contacted. Because no expert teams for the care of TS individuals currently exist, the endocrinological and surgical treatment of the individual is influenced predominantly by reasons unrelated to the medical needs of the patient, such as the attitudes of a particular doctor and the availability of an examination or treatment. Even in instances where the mental health professional does approach transsexuality in a contemporary manner, he or she has nowhere to direct the patient for surgical treatment other than foreign facilities (most often in Serbia). Furthermore, Croatian health insurance does not cover the costs of treatment that such a diagnosis requires, which means that TS persons must rely solely on their own financial means to receive necessary medical treatment.

Together, these circumstances surrounding the medical care of TS persons in Croatia cause most individuals to resign themselves to isolation and not seek help. Clinical experience has shown that most TS individuals eventually give up considering sex change [15] and come up with more or less satisfactory ways of life that do not include surgery. The results of research conducted with 16 transgender persons demonstrated that a majority of individuals were afraid that their disorder would be discovered that they often stopped considering surgery as an option and received little support from their family, partner, and social environment [15]. In the previously described Croatian context, this is undoubtedly the result of the unavailability of adequate medical care, a lack of legal regulation, and the negative attitudes of not only the general population, but also the medical practitioners responsible for providing care to TS persons.

Currently, there are two civil society organizations (CSO) in Croatia aiming to address this issue: one that has been operating for the past two years that primarily focuses on activism and advocacy and the other is providing psychological counselling. This latter organization has been operating for 15 years and aims to provide services consistent with the Standards of Care (SOC) of World Professional Association for Transgender Health (WPATH) for psychosocial care. The data presented in this paper were collected in a psychological counselling centre located in the capital city of Croatia, Zagreb. This centre is staffed by psychologists who work on a primarily voluntary basis and provide initial psychodiagnostic assessment and counselling to TS individuals and their families. For people undergoing gender transition, psychological monitoring is organized through all phases of medical treatment. Over the years, psychologists have established an informal network of other professionals in Croatia (endocrinologists, psychiatrists, general practitioners, and gynecologists) that are transfriendly and to whom TS individuals can be referred. For SRS, patients are referred to medical facilities outside of Croatia and most commonly to Serbia, where personal contacts have been made with experts in transsexuality. Support groups have also been organized in the counselling centre.

Despite these challenging circumstances, some gender dysphoric individuals in Croatia persist in achieving the goal of making their own physical characteristics match their gender identity and have continued to live in Croatia after surgery. This paper will examine the psychosocial adjustment of six transsexual individuals living in Croatia following SRS.

The paper aims to depict the factors contributing to psychosocial adjustment despite the poor social and medical circumstances faced by transgender individuals. A combination of quantitative and qualitative self-report methods was used, employing well-recognized and established questionnaires to assess the mental health status of participants and a series of open-ended questions to gain insight into the complex experience of psychosocial adjustment following SRS in Croatia. Undoubtedly, the described situation in Croatia is also the case in other countries. As such, understanding the factors and processes that support mental health in marginalized populations has the potential to make an enormous impact on the quality of life, safety, and health-seeking behaviors of individuals in both Croatia and other contexts.

## 2. Methods

### 2.1. Participants and Procedure

In accordance with the qualitative and exploratory nature of the study, in which the subjective experience of adjustment following SRS was examined, a purposive sampling strategy was employed to select participants meeting several study criteria. The intended sample of our study was all TS persons in Croatia who underwent SRS in the last 15 years and who underwent psychologist-supervised transition in the centre for psychological counseling in Zagreb prior to surgery, a program provided entirely outside of public health care. In the last 15 years, 42 TS persons have sought support at the centre, 8 of whom have undergone SRS. While there are certainly more people living in Croatia who have completed the SRS process in this period, they did not do so within the context of standardized or supervised health care and, as such, no corroborating valid data exists. Of the 8 individuals identified through the counseling centre, 6 agreed to participate in our study. One MtF individual who underwent surgery in Croatia 15 years ago and currently lives abroad declined the invitation to participate in the study because of her negative experience with the Croatian health care system. Another FtM individual appears to have emigrated out of Croatia and is no longer in contact with people in the* trans* community, and was therefore unavailable. In Table 1, demographic data of the sample c is presented.

### 2.2. Data Collection

The method of data collection was chosen based on the specificity of the sample. Namely, of the six participants taking part in the research, 3 lived in the same location as the researchers, while two lived in other cities in Croatia and one outside of Croatia. This made data collection via in-depth interview impossible and made online data collection a necessity. For this reason, a questionnaire was created using Survey Monkey. The first section of the survey consisted of standardized questionnaires and questions pertaining to demographic data, while the second section included a series of open-ended questions divided in 4 themes: (1) the decision-making process regarding SRS; (2) social and medical support during the SRS process; (3) experience of discrimination and stigmatizing behaviors; (4) psychosocial adjustment after SRS.

A letter of invitation and a brief description of the research, along with a link to the online questionnaire, were sent via E-mail to all 8 available individuals who met the study criteria. Six participants completed the questionnaire. As previously mentioned, one MtF participant declined participation due to an extremely negative experience with SRS in Croatia.

### 2.3. Data Analysis

The results of the standardized questionnaire were calculated for each participant and compared to Croatian norms.

In order to analyze open-ended questions, a data input table with all responses was constructed, after which observations, reflections, and remarks were noted. Following this analysis, common themes or patterns, as well as commonalities and differences in the participants' experiences, were identified.

### 2.4. Instruments

#### 2.4.1. SF-36 Health Survey Croatian Version

The Croatian version of SF-36 Health Survey is a multipurpose, short-form health survey which consists of 36 questions [19]. Each of the questionnaire items refers to one of the following eight health indicators: physical functioning (10 items); role-physical, a limitation in performing important life roles due to physical health (4 items); bodily pain (2 items); general health (5 items); vitality (4 items); social functioning (2 items); role-emotional, a limitation in performing important life roles due to emotional problems (3 items); mental health (5 items). The total result is most often shown in the form of an 8-point profile that individually represents each measured aspect of health on a scale from 0 to 100. On all scales, higher scores indicate better subjective health. The internal consistency of the SF-36 scales ranged from 0.78 to 0.94 [19].

#### 2.4.2. CORE-OM—Croatian Version

The Croatian version of the CORE-OM (Clinical Outcome in Routine Evaluation—Outcome Measures) [20] contains 34 items on which clients record how often they have felt a certain way during the past two weeks (0: never, 1: very rarely, 2: sometimes, 3: often, and 4: almost always). These items refer to four dimensions: subjective gain (4 items); problems/symptoms (12 items); daily functioning (12 items), and risky behavior (6 items). Individual results, results on risk-free items (all items apart from those dealing with risky behavior), and overall results are presented as an average result (the overall result divided with the number of items on a particular scale or dimension). Higher overall or individual results reflect a greater number of problems and higher levels of distress. This also applies to the dimension of subjective gain, in which a higher score indicates a lower level of gain and more difficulties in this area. Research has indicated that the CORE-OM has satisfactory reliability, expressed through a measure of internal consistency ranging around 0.90 for the overall scale and between 0.70 and 0.90 for individual dimensions [21, 22].

#### 2.4.3. The Depression Anxiety Stress Scale

The depression anxiety stress scale (DASS-21) [23] is a self-assessment measure that examines the frequency and prominence of negative emotional states (depression, anxiety, and stress) during the last seven days in both psychiatric patients and healthy population. The scale consists of 21 items divided into three subscales: the depression scale (7 items), the anxiety scale (7 items), and the stress scale (7 items). Participants are asked to assess each of the given symptoms on a scale from 0 to 4 based on their experiences during the previous week. Reliability coefficients of the Cronbach alpha (*α*) type were 0.82 on the depression scale, 0.84 on the anxiety scale, and 0.83 on the stress scale [24].

Six TS participants took part in the present study: 3 FtM and 3 MtF, ranging in age from 24 to 42 years. Five participants experienced an early onset of transsexuality, with the remaining participant experiencing late onset. Educational achievement for all participants is at the graduate level, and five participants are currently employed (the remaining participant is still a student). Three of the participants are single and three are in a relationship, with one participant living with their partner. Five participants are homosexual according to natal sex, and one is heterosexual. All participants underwent SRS outside of Croatia: four in Belgrade, Serbia (all FtM and one MtF), and two in Phuket, Thailand. All SRS were performed in the period between 2009 and 2013.

## 3. Results

Individual results on the SF-36, DASS-21, and CORE-OM questionnaires are presented in Table 2. On the DASS, all participants achieved results that fell within the category of normal mood variations. This finding is also true for both the subscales and the overall results on the CORE-OM questionnaire, where all the results fall under gender specific cutoff scores. On the SF-26, three participants achieved scores on some of the scales that fell below the average normative results for age and gender; one MtF (33) participant achieved a lower score on the mental health scale only; the oldest MtF (42) participant achieved lower than average results on six SF-36 scales; one FtM (33) participant achieved lower than average scores on five SF-36 scales.

As described previously, participants were asked a series of open-ended questions related to the decision-making process regarding transition and sex reassignment surgery, their experience of discrimination due to gender dysphoria, social support during the process of transition, and psychosocial adjustment after surgery. Responses to these questions were analyzed using the thematic, qualitative approach described previously.

### 3.1. Deciding to Undergo SRS

All participants in the study are transsexual persons who expressed a pronounced desire for sex reassignment surgery. Other options were not considered to be plausible alternatives by any of the participants.

*Transition without surgery was never an option for me as I would have considered such a transition unsatisfactory and incomplete. So, it was right from the start that I saw surgery as the final step in making my body complete in the male sense. (FtM, 24)*

*I would never have been content without surgery, would never have been able to have this lifestyle and I would have been unhappy with my body. Surgery was the only way for me to reach contentment and happiness and to live my life to the fullest. (MtF, 34)*

*There was no alternative because that would have meant a continuation of suffering. I ascertained that it was the only way I could shed transsexuality and have a better quality of life. (FtM, 33)*

However, although the decision to undergo SRS was never in question for any of the participants, the decision-making process itself was hindered by various aggravating circumstances including a fear of how those around them would react and, for some participants, financial difficulties.

*I was somewhat afraid of how those around me would react as only a very small number of people knew of my transition, but I hoped for the best. The people who did know of my transition were those closest to me and were supportive, so that made it a bit easier to expect others to accept it too. (MtF, 34)*

For some participants, the period preceding surgery was marked by various fears related to the outcome of the operation (in terms of functionality and aesthetics) and a fear of postoperative complications. The fear that one's health would suffer and that a dependence on others would be the result was also a source of worry for some TS persons. Because there are currently no SRS centres in Croatia, TS individuals are forced to decide at which foreign centre they will undergo surgery. In order to make this decision, participants reported primarily gathering information informally (via the Internet and from other TS persons), albeit using a very serious and systematic approach. In selecting the right foreign centre, participants considered three things to be particularly important: the quality of services rendered based on the experience of those who had already undergone surgery (this information was mostly gathered online), the individual's ability to cover financial costs, and the personal care provided at a particular centre. The reputation of the surgeon chosen to perform the operation was also of great importance.

*The fact that the doctor I chose was very well renowned and respected in this area and that his method of performing this surgery was considered one of the best was, for me, the most important factor in deciding on where I was going to have the surgery. (MtF, 34)*

*I considered the relative closeness of the centre, the price and very good results to be most important. Actually, the results of the surgery were some of the best I'd come across during my research online. (FtM, 24)*

Most participants underwent SRS within three years following the time at which transsexuality was formally identified. However, for some participants, the period between deciding to undergo surgery and actually undergoing SRS was as long as 10 years. For the most part, these lengthier periods can be attributed to financial issues and other circumstances within the lives of individual participants (such as completing college studies, family problems, and working conditions). The fact that endocrinological treatment also needed to be arranged and provided outside of Croatia, which included finding a doctor, ensuring sufficient funds for covering the costs of this process, and addressing various organizational problems, also contributed to the length of this period.

All participants expressed very high satisfaction with their decision to undergo SRS. The main source of this satisfaction stems from the experience of feeling whole and a new harmony between their psychological and physical selves. Also noteworthy was participants' reports of a growth in self-esteem as a result of having a physical body that matched their gender identities.

*I finally feel like a man who's complete. I'm satisfied simply by knowing that I no longer have female genitalia and that it looks completely male. Certainly, it's also a great pleasure to be able to pee standing up. (FtM, 24)*

*Everything turned out differently than what I had been expecting because I had expected to go through at least some uncomfortable situations, but everything turned out great. (FtM, 37)*

Participants also spoke about the difficulties encountered following SRS and upon return to one's social environment, which were largely related to the way in which society reacted to them and to an internalized transphobia that manifested itself through feelings of guilt.

*The feeling that you've committed a crime and gotten away with it never leaves you. I don't know how real a problem this is because everyone has so far treated me well. (FtM, 33)*

MtF individuals reported having problems in finding an emotional partner as well as experiencing fear about having to inform their male partner about their transition process.

*My past is always following me and it does catch up with me in the end. It isn't that I'm ashamed of it, but that I don't want everyone I meet knowing about it because I have the feeling that they perceive me differently if they find out right away. The same thing happens when I'm in a romantic relationship because I'm always worried about how to tell the person and about how they'll react when I tell them. (MtF, 34)*

None of the FtM participants mentioned any problems in partnership.

In the period following SRS, four participants reported being sexually active (two FtM and two MtF) and were sexually active exclusively with people of the opposite sex. For the most part, participants agreed that sex was of moderate importance.

*The importance level I attach to sex changes from one period to the next; sometimes even I can't be sure how important it is. I like it, but considering how frequently I have it I don't think it's too important to me. (FtM, 24)*

Almost all participants reported feeling pleased by how well SRS has allowed them to function sexually. For FtM participants, satisfaction was derived from the fact that they could now be sexually aroused with their newly formed genitals without feeling shame and being uncomfortable, by the appearance of their penis and by the fact that they could achieve erection and could urinate standing up. For the most part, MtF participants reported being most pleased by the appearance of their genitals.

*I'm satisfied by the anatomical appearance. (MtF, 33)*

*When I started having sex after the surgery I was more than pleased with how everything works—from the compatibility of the genitals, vaginal lubrication and ability to achieve orgasm. (MtF, 34)*

When participants were asked about their expectations regarding sexual functioning following SRS, most participants reported that their expectations had been fulfilled. However, one FtM participant reported expecting a bigger penis, while another reported expecting that he would be able to ejaculate. In addition, an MtF participant reported expecting to derive greater sexual satisfaction from stimulation.

Participants were also questioned about their experience with certain sexual activities following SRS. All participants reported engaging in masturbation. During this activity, none of the FtM participants reported having any sexual difficulties or problems. Two MtF participants complained about decreased sexual desire and one also noted that her experience of orgasm was now less intense. Participants reported that these problems did reduce satisfaction with their own sexual functioning.

*I'm not particularly satisfied. My orgasms are “shallow”, while my desire for sex is much lower. (MtF, 34)*

As far as other sexual activities are concerned, four participants reported experiencing penetration following SRS. While two participants reported having had no problems, another two individuals reported problems in relation to penetration. Specifically, one FtM participant described having a problem with incomplete penetrative sex even with the help of purchased aids, while another MtF participant reported problems with orgasm and pain during penetration.

*It takes me longer to achieve orgasm. Also, sometimes penetration that is too deep hurts. (MtF, 33)*

Of other sexual activities, half of the participants reported experiencing petting, while experiencing oral and anal sex was reported by only one participant (MtF) following transition.

### 3.2. Discriminatory Behavior

In the first part of the questionnaire, participants were asked about the sources and types of discriminatory behavior they may have experienced during the SRS process. Two participants (FtM) reported never experiencing discriminatory behavior of this sort.

*I have never experienced discriminatory behavior in any situation (FtM, 37)*

Amongst participants who did report experiencing discrimination, such behavior was reported to come mainly from colleagues in the workplace and from administrative staff with whom they had come into contact during the SRS process. The incidence of such behavior was the lowest with friends and medical staff with whom they went through the SRS process.

It is important to note that all reports of discriminatory behavior of medical staff towards participants came exclusively from Croatian medical staff. In these instances, participants reported negative experiences such as deprecation, lack of understanding, and unwillingness to provide medical help.

*I have had contact with so-called surgeons from Zagreb who think it's their prerogative to say who's male and who's female. (MtF, 42)*

*An endocrinologist from Zagreb who'd heard I wanted a sex-change started yelling at me that I wanted to have the surgery only so I could fool men. (MtF, 33)*

Other discriminatory behaviors are reported by participants including receiving insults and being ignored or provoked. Participants also reported experiencing instances in which people were simply stunned.

### 3.3. Social and Medical Support during the SRS Process

In the second part of the questionnaire, the degree to which participants were satisfied with the support provided to them during the SRS process by family members, friends, colleagues, other TS individuals, and professionals (psychiatrists, psychologists, surgeons, endocrinologists, and general practitioners) was examined.

For four participants, the main sources of support during the SRS process were family members and friends. They reported that such support and acceptance from people they have known and have cared for their entire lives was very important to them.

*Family, friends, my girlfriend—almost everyone around me. Their support could be seen in their understanding and care, and that's what was most important to me. It's very important to me for people I care about to understand and love me. (FtM, 37)*

*My immediate family had several different functions to perform during that process—they helped me financially, offered me support and care after the surgery but were, at the same time, openly critical of the entire process and, by doing so, reflected how others around me might react. (FtM, 24)*

*My family was both a source of financial support and my harshest critics. I knew that they wouldn't abandon me, but they were skeptical. (FtM, 33)*

*My family and friends were a great source of support. My parents and sister were with me during the entire time of my recovery; they helped me, took care of me and cheered me on when it was hardest. (FtM, 24)*

Friends were most often the first people with whom participants confided in and from whom they asked for understanding and acceptance. For most participants, the role of friends during the SRS process was of an emotional nature rather than an instrumental one.

Participants with a partner reported that their partners were a great source of support during the SRS process, in both the emotional and the instrumental sense.

*My girlfriend was a source of emotional support and instilled a sense of self-confidence in me in terms of being a man. She also took care of me during my recovery. (FtM, 33)*

*Friends and others around me offered me support by accepting me completely. (MtF, 42)*

Two MtF participants reported that other TS individuals offered the most support, noting that these individuals were those who could completely understand what they were going through and who were familiar with the fears and worries they had experienced before surgery. Moreover, other TS individuals were a valuable source of information.

*The support of other TS individuals could be seen clearly in their understanding of my situation. (MtF, 34)*

On the whole, findings indicate that friends, partners (in instances where participants had one), and other transgender individuals were the greatest sources of support during the SRS process. All participants additionally noted how valuable the support of other TS persons was for them.

*Yes, my fears were unfounded. Everyone who found out about my sex-change was supportive after recovering from the initial shock of it. (MtF, 34)*

Participants were also asked about the support level offered by experts with whom they came into contact during the SRS process. Findings indicate that, for the most part, the participants' surgeon and psychologist were the most supportive professionals during this process. Participants reported feeling that the support received from the surgeon was of particular importance, where the surgery itself is perceived as a very delicate matter whose outcome is not only medically but also psychologically relevant. It was also very important to participants that the surgeon possesses a high level of expertise. Together, this expertise and support was reported to have had a great psychological impact on participants, allowing them to feel supported and accepted and to have confidence in the outcome of the surgery.

*My greatest source of support was my surgeon as that's the most important phase of the transition. I trusted him because he was an expert, was accommodating and would adapt to his patient. (FtM, 33)*

When speaking about psychologists, participants reported that support from psychologists was most clearly visible in the understanding they offered and in their assistance in solving problems that preceded the surgery (i.e., the process of deciding whether to undergo surgery itself).

*My greatest source of support was my psychologist because of her realistic approach to the problem. (MtF, 42)*

The manner and extent to which self-help TS support groups were important to participants were also examined. Participants reported that this type of support had two functions: one emotional and one instrumental.

More specifically, participants reported that other TS individuals were able to understand the emotional experiences they had gone through, help them achieve self-acceptance, and offer them an opportunity to discuss their SRS-related fears openly and without shame. In addition, other TS individuals were reported to be a valuable source of information and shared experience in helping participants deal with the problems they were facing. In these settings, other TS individuals provided information and support while deciding whether to undergo the SRS process, including information about specific medical centres and experts offering SRS.

*The prime advantage of it is that you're swapping experiences and discussing the subject with people who are in exactly the same situation as you. (MtF, 33)*

Interestingly, participants also made note of the potential disadvantages of this type of support. Namely, they reported feeling that being part of a support group and having intense contact with other TS individuals in various stages of their own transition can make one feel eager to speed up one's own transition. Consequently, one risks entering into the transition process without truly considering all the advantages and disadvantages of specific stages of the process (e.g., hormonal therapy).

*After they've started their own transition process, some people meet someone who's already further along than they are, start to feel invigorated and start speeding their own process along without giving everything the necessary thought. (MtF, 37)*

*The desire of new members to achieve their goals over-night after meeting those who've already gone through the process. (MtF, 34)*

When speaking about the support of other TS individuals, participants also noted that this source of support was relevant only during the transition process and that they often lost contact with other TS persons after successfully completing their own process of transition. However, this situation did not apply to instances in which more long-term friendships had been formed. Participants reported feeling that, following completion of the SRS process, they no longer perceived themselves as people with problems or a disorder and that, as such, they no longer needed the support of other TS individuals.

*It was only during transition that the support of other TS people was relevant to me as I wanted to hear their experiences and see the results of their transition. I no longer need it nor am I interested in participating in such groups, apart from keeping in contact with some people with whom I've become long-term friends. (FtM, 37)*

### 3.4. Recommendations for TS Persons in Croatia

Participants were asked to express their opinion as to why most TS individuals from Croatia eventually stop considering SRS and, in light of this fact, what advice they would give other TS persons in Croatia.

All participants agreed that financial issues were the main reason for a person to reject SRS and that lack of expert and family support were additional contributing factors. In general, they advised that TS individuals should be completely certain of their decision before undergoing SRS, be well informed, and have realistic expectations and patience. They also noted that living a fulfilled life is important.

*I would advise them to get as informed as possible on the nature of the surgery, to know what they can expect and to have a plan ready for each outcome. You have to be very patient and there is a possibility that not everything will work out as it should the first time around (for instance, the results of a surgery may need to be corrected). Also, getting the surgery isn't the end of it—you have to go back to your “regular life”. What I mean is that it's important not to give up on your education, job and friendships because that's all equally important for the process to succeed. Everything needs to be happening at once. I'd also advise them to be less afraid of how those around them will react because what's most important is how free and happy they are with themselves. (FtM, 33)*

## 4. Discussion

The aim of this study was to depict the factors that contribute to the psychosocial adjustment of transsexual individuals who have undergone the gender transition process (including SRS) in a country in which the social environment is intolerant towards gender nonconformity.

Transsexuality is a universal phenomenon that affects individuals in all cultures. The standards of care for persons experiencing gender dysphoria and transsexuals as a subgroup relying on medical intervention regulate the provision of care in countries with a developed liberal democracy and high social standard. These are primarily western European countries, the USA, and highly developed countries in Eastern Asia. In these countries, gender identity is considered from a human rights perspective and mental health and medical professionals are obligated to approach the provision of health care for transsexual, transgender, and gender-nonconforming persons according to the Standards of Care of WPATH [25]. However, even some contexts providing gender reassignment treatment fail to provide appropriate and accessible treatment to all* trans* people [26]. Recent research in countries in the European Union indicates that TS persons are neither adequately supported nor given sufficient access to treatment because medical professionals lack the necessary knowledge in trans-related health care and health care systems refuse access to funding for treatment [26].

In countries where gender dysphoria is viewed not as gender variation but as a psychiatric disorder, transsexual persons are confronted with an additional set of challenges due to lack of regulation, lack of professional expertise in the area of* trans* health, and difficult pathways for accessing and arranging treatment. From 2013, Croatia is a full member of the European Union and possesses legal avenues for the process of legal name and gender change following sex reassignment surgery. However, Croatia has no national guidelines or legal, ethical, and professional recommendations for the organization of care for transsexual persons and no medical facility offering SRS. Attitudes towards sexual minorities and transsexual persons are mainly negative [17]. In such a context, gender nonconformative persons are exposed to high minority stress [27, 28]. Furthermore, transsexual persons are also exposed to stress stemming from contact with health professionals whose behavior is not based on evidence-based medical recommendations but on personal belief systems or religious or cultural prejudice. Undoubtedly, psychosocial adjustment following SRS in such a social context is not without various unique and significant obstacles. The present study aimed to examine this difficulty and not yet researched experience.

The study included 6 transsexual persons (3 MtF and 3 FtM) who had completed the process of gender transition. All medical procedures for gender reassignment were organized outside the country, while psychological support was provided in Croatia. All subjects underwent SRS, reporting a view that it was their only option.

Data collected through standardized mental health questionnaires indicated that all participants are in a good mental state. Three participants achieved somewhat lower results in the sections examining quality of life, which can be attributed to their general physical condition. In one case, an MtF (42) participant achieved low results on six of the SF-36 health survey scales, which can be explained by the fact that the participant underwent SRS only three months prior to completing the questionnaire and was still dealing with various postoperative consequences. For this participant, none of the results achieved fell below the 25th centile and therefore do not represent an extreme deviation from the norm. The fact that this should be regarded as a reflection of physical rather than psychological symptoms is supported by the elevated results achieved by this participant on standardized questionnaires measuring psychological well-being (CORE-OM and DASS). For two other participants, lower results on the quality of life questionnaire might have been due to internalized transphobia, demonstrated by their responses given later in the questionnaire as well as the results achieved on other mental health scales.

The qualitative results similarly suggest generally good psychological adjustment amongst all participants. All participants reported being satisfied with their decision to undergo SRS and with the way the operation was performed. They reported being highly motivated to match their physical bodies with their gender identities and had decided to undergo the SRS procedure despite unfavorable psychosocial circumstances. Although they expressed certain fears before the surgery, participants asserted that these fears were not realized. In general, participants are well regarded in their social surroundings and are highly functional. The difficulties mentioned by participants related either to sexual functioning (noted by some participants) or to internalized transphobia, but none of these difficulties endangered their mental health or overall functioning in various social roles. It should be considered that some TS individuals will need psychological support after SRS.

A recent meta-analysis indicated that transition leads to a significant increase in quality of life for 80% of TS persons and a decrease in psychological disturbances in cases where such disturbances had been present before transition [29]. Conversely, the determination of morbidity and mortality in a cohort of TS persons from Sweden demonstrated that post-SRS transsexual individuals are at a considerably higher risk of mortality, suicidal behaviour, and psychiatric morbidity than the general population [30].

The results of the present study confirm the generally stable and favourable function of TS individuals following SRS. Prior research examining the outcomes of SRS suggests that factors contributing to favorable outcomes include good social support, absence of psychopathology, good surgical results, and satisfaction with physical appearance [31, 32]. This was similarly reflected in the results of the present study, where participants reported these factors in their responses concerning postsurgical adjustment. Namely, participants reported having good social support and were also very pleased with the surgical results and the manner in which they were treated by the medical staff performing the surgery. This, in turn, greatly influenced their feelings of satisfaction with the procedure. Moreover, several other protective factors were present amongst the participants, where all participants held a high SES, had good premorbid functioning [15], and, most importantly, were highly motivated to align their physical bodies with their gender.

The challenges that the participants faced were numerous and included a lack of local standards of medical care, organizational difficulties related to the transition process (e.g., finding an endocrinologist, a surgeon and hair removal professionals tolerant towards the needs of TS individuals), legal problems, lack of financial means needed to cover medical costs (all participants needed to pay for endocrinological and surgical treatment themselves), and discrimination. For the participants in this study, the lack of organized care for TS persons in Croatia contributed to a lengthier period between when they commenced of the transition process and when they underwent SRS than that is common in countries with standards of care [26]. Previous research carried out in a number of EU countries has suggested that a belief, amongst health care professionals, that individuals seeking gender transition suffer from a mental illness is a significant factor contributing to the inappropriate provision of care [26]. In these instances, TS individuals are most often referred to psychiatric treatment instead of accessing the medical care necessary for supporting transition. In Croatia, where high level of heteronormativity amongst medical professionals is the norm, it is nearly impossible for TS individuals seeking support to receive appropriate care through the public health system. Instead, the participants in the present study sought support through the psychological counseling centre, where psychologists knowledgeable in the specific needs of TS individuals provide counseling services in accordance with the standards of care. Access to appropriate medical care is gained through informal networks established between the counseling centre and local and international physicians with expert knowledge and experience in supporting TS individuals. Although this process typically takes a somewhat longer period of time, all care is provided in accordance with the standards of care.

Arguably, it is possible that this extended period is one factor that positively influenced the participant's adjustment following SRS. In addition, the mere passage of time might have had a positive influence on the adjustment of the participants' families and social surroundings. Participants consistently reported that the support of family members, friends, and partners was very relevant to them. The importance of receiving support from those close to TS individuals has also been highlighted in previous research [32]. In light of these findings, it is reasonable to expect that family and friends themselves are also in need of expert psychoeducation and support. A study conducted on a representative sample of Croatian citizens indicated that more than 60% of participants consider transsexuality to be abnormal and would seek support from a psychiatrist if their child expressed transsexual feelings. Approximately one-fourth of respondents reported that, in this situation, they would throw the transsexual family member out of their home [17]. Arguably, the prolonged period preceding SRS experienced by our participants enabled both TS individuals and their families to more successfully adjust. During this period, participants had sufficient time to carefully plan their professional and social life following SRS, had participated in psychological treatment for a longer period, and had realistic expectations about the transition process and postsurgical adjustment reinforced.

Another factor potentially influencing the adjustment of participants in the Croatian context stems from a cultural-linguistic specificity, where the highly gender-polarized national culture is notable in the Croatian language itself. The Croatian language belongs to group of grammatical gender languages, in which recent research in language and gender has recognized that the language not only reflects, but also constitutes gender [33–35]. In this group of languages, which constitute approximately one-fourth of the world's languages, nouns are classified according to grammatical gender and every noun holds a single gender value. Therefore, in the Croatian language (as with all Slavic and some Roman languages), the representation of oneself (i.e., self-perception and self-labeling), as well as communication with others, is impossible without the reflection of gender. For example, I am hungry in English can be stated as* Ja sam gladna* (feminine) or* Ja sam gladan* (masculine), depending on whether the speaker is male or female. Similarly, in Croatian conversation, the question, “Are you hungry?” is posed as* Jesi gladna?* (feminine) or* Jesi gladan?* (masculine). As a result, a binary model of gender is present in nearly every sentence and, as such, it becomes very difficult to think about oneself and of others without this dualism. Furthermore, it is much easier to think and speak about oneself and to be perceived by others, if one's appearance is congruent with one's gender identity. This linguistic characteristic becomes significant in light of its consequences for the care of TS individuals. Specifically, the SOC recommendation for health professionals and families to respect the desired gender of a TS person in all means of communication becomes nearly impossible to be implemented in the Croatian context, where a TS individual will be referred to by his/her present gender appearance in all social settings. As a result, the period of time required by a TS individual and his or her social network to adjust to the* trans* identity is probably much longer in this culturally linguistic context. This linguistic duality is further reinforced by the collectivist nature of Croatian society. In such societies, it is common for gender identity to be based on a binary social construct [36]. It might be further hypothesized that these sociocultural circumstances make it easier to be transsexual (as opposed to any other sorts of transgendered person). This hypothesis should be examined in further research.

At first glance, these results appear to be a positive depiction of the psychosocial adjustment of TS individuals in Croatia. However, several specificities of the present study and the recommendations that might be derived need to be noted. Namely, the participants in this research represent a highly specific sample of persons who underwent the SRS process in a country without organized standards of care but who received continued psychosocial care during their transition process. Although this care was delivered in a CSO context and was a result of the personal enthusiasm of individual psychologists, it was conducted under the Standards of Care, 6th version [37] active at the time of care delivery. Although the present study did not examine the adjustment process of individuals who do not receive such care but are forced to fend for themselves, it seems reasonable to assume that the care afforded to the TS persons participating in the present research contributed to their adjustment well after surgery. The data collected in the qualitative part of the research indicated that participants felt it was very important to be “well informed,” “patient,” and “to not ignore other areas of life such as education and friendship.” These aspects of adjustment were all directly addressed during psychological counseling. The results about sexual functioning after SRS emphasize the importance of sexual counseling for even better psychosocial adjustment of TS persons.

In countries that do not offer organized support to TS persons, self-organized support groups are a common way of spontaneously organizing peer support for TS persons [38]. The results of the present study suggest that, in addition to being invaluable for creating a sense of belonging and understanding amongst TS persons and being a valuable source of information, these support groups represent a potential hazard by accelerating transition without expert support. If this potentially negative effect is to be minimized, it is therefore important that such groups are not the sole source of support in such countries. The results further suggest that it is important to offer organized and guided support outside of the health care system and for experts to be educated and informed about the current standards and recommendations of WPATH.

The main limitation of the present study is the small number of participants. While the findings have allowed for some insight into the individual experiences of TS persons in Croatia, the size of the sample does not allow for any generalization. Another limitation arises from the fact that one of the study authors was also the psychologist providing psychosocial counseling to all participants. Although this author did not directly collect the data, it is possible that participants wished to provide “good” data. A final limitation of the study arises from the data collection method used. Because of the varying geographical locations of the participants, face-to-face interviews proved to be difficult to be organized, even those via Skype. However, the electronic method of data collection applied did have advantages in that it allowed the participants to answer questions anonymously on their computers, a factor which might have enabled them to answer more honestly and frankly.

## 5. Conclusion

As the first study in Croatia conducted with persons who have undergone gender reassignment, the results of the present research confirm the importance of individual factors in the psychosocial adjustment of transsexual individuals following SRS in a country whose social environment is hostile towards gender nonconformity. Despite the unfavorable circumstances currently existing in Croatian society and health care, participants demonstrated stable mental, social and professional functioning, and a relative resilience to minority stress. The results also demonstrate the potentially protective role that a lengthier process of transition plays in countries such as Croatia. Arguably, the longer duration of gender transition strengthens individual resolve while also allowing both TS individuals themselves and those around them to better adjust to sex transition amongst TS persons. The absence of a robust body of research on transsexualism in Croatia limits knowledge about relevant factors contributing to the psychosocial adjustment of TS individuals living in country with predominantly negative public attitudes and no standards of care. Further research is needed to illuminate these results by creating one multicentric study which will follow all TS persons referring to psychological and medical care.

The findings of the present study also strongly confirm the biopsychosocial model and basic principles of the SOC, which place strong emphasis on a culturally sensitive approach. The most recent edition of the SOC [25] contends that even health professionals operating in contexts in which limited resources and training exist can effectively apply the core principles of care. These principles include maintaining a respect for patients, providing care that affirms gender identity and reduces the stress of gender dysphoria, being knowledgeable about the health care needs of TS individuals, matching the treatment approach to the needs of the individual, enabling access to appropriate care, and offering support for individuals within their families and communities [25]. The findings of the present study support this argument and confirm that, even in an intolerant context with few outlets for appropriate care and support for TS individuals, it is possible to effectively assist TS individuals throughout the transition process and to ensure the conditions for successful psychosocial adjustment following SRS.

## Figures and Tables

**Table 1:** Demographic characteristics of participants (*N* = 6).

Transition	Age	Onset	Education	Employment	Relationship status	Attracted to:	Operation performed in	Year of operation
FtM	24	Early	Student	Student	Single	Female	Serbia	2013
MtF	34	Late	M.A.	Employed	Single	Female	Thailand	2010
FtM	37	Early	M.A.	Employed	In a relationship	Female	Serbia	2010
MtF	33	Early	M.A.	Employed	In a relationship	Male	Thailand	2009
MtF	42	Early	M.A.	Employed	Single	Male	Serbia	2013
FtM	33	Early	PhD	Employed	Cohabitation	Female	Serbia	2012

**Table 2:** Results on CORE-OM, SF-36, and DASS-21 for each participant (*N* = 6).

Transition	Age	SF-36								CORE-OM					DASS-21		
		PF	RP	RE	SF	MH	EV	P	GH	F	P	SWB	R	ALL	DEP	ANX	STRESS
FtM	24	100	100	100	100	84	80	90	95	0.08	0.42	1.00	0.00	0.29	1.00	3.00	1.00
MtF	34	100	100	100	100	100	75	80	85	0.83	0.25	1.50	0.00	0.56	1.00	1.00	2.00
FtM	37	100	100	100	100	88	85	90	85	0.50	0.25	1.00	0.00	0.38	0.00	0.00	0.00
MtF	33	100	100	100	88	68*	70	80*	77	0.75	0.75	1.25	0.00	0.68	3.00	0.00	4.00
MtF	42	75*	100	67*	63*	60*	60	70*	57*	0.92	1.42	1.50	0.17	1.03	5.00	3.00	6.00
FtM	33	100	100	33*	100	68*	50*	80*	72*	0.58	1.00	1.25	0.17	0.74	3.00	0.00	3.00

Note.  *Results lower than normative average score for gender and age group. SF-36 scales: PH: physical functioning; RP: role physical; RE: role-emotional; SF: social functioning; MH: mental health; EV: vitality; P: bodily pain; GH: general health. CORE-OM scales: F: functioning; P: problems; SWB: subjective well-being; R: risk; ALL: total score. DASS-21 scales: DEP: depression; ANX: anxiety; STRESS: stress.
